# Dynamic but discordant alterations in zDHHC5 expression and palmitoylation of its substrates in cardiac pathologies

**DOI:** 10.3389/fphys.2022.1023237

**Published:** 2022-10-05

**Authors:** Alice Main, Andri Boguslavskyi, Jacqueline Howie, Chien-Wen Kuo, Aileen Rankin, Francis L. Burton, Godfrey L. Smith, Roger Hajjar, George S. Baillie, Kenneth S. Campbell, Michael J. Shattock, William Fuller

**Affiliations:** ^1^ Institute of Cardiovascular and Medical Sciences, University of Glasgow, Glasgow, United Kingdom; ^2^ School of Cardiovascular and Metabolic Medicine and Sciences, King’s College London, London, United Kingdom; ^3^ Flagship Pioneering, Cambridge, MA, United States; ^4^ Division of Cardiovascular Medicine, University of Kentucky, Lexington, KY, United States

**Keywords:** palmitoylation, hypertrophy, heart failure, ZDHHC5, cardiac muscle, depalmitoylation, ion transporter

## Abstract

S-palmitoylation is an essential lipid modification catalysed by zDHHC-palmitoyl acyltransferases that regulates the localisation and activity of substrates in every class of protein and tissue investigated to date. In the heart, S-palmitoylation regulates sodium-calcium exchanger (NCX1) inactivation, phospholemman (PLM) inhibition of the Na^+^/K^+^ ATPase, Nav1.5 influence on membrane excitability and membrane localisation of heterotrimeric G-proteins. The cell surface localised enzyme zDHHC5 palmitoylates NCX1 and PLM and is implicated in injury during anoxia/reperfusion. Little is known about how palmitoylation remodels in cardiac diseases. We investigated expression of zDHHC5 in animal models of left ventricular hypertrophy (LVH) and heart failure (HF), along with HF tissue from humans. zDHHC5 expression increased rapidly during onset of LVH, whilst HF was associated with decreased zDHHC5 expression. Paradoxically, palmitoylation of the zDHHC5 substrate NCX1 was significantly reduced in LVH but increased in human HF, while palmitoylation of the zDHHC5 substrate PLM was unchanged in all settings. Overexpression of zDHHC5 in rabbit ventricular cardiomyocytes did not alter palmitoylation of its substrates or overall cardiomyocyte contractility, suggesting changes in zDHHC5 expression in disease may not be a primary driver of pathology. zDHHC5 itself is regulated by post-translational modifications, including palmitoylation in its C-terminal tail. We found that in HF palmitoylation of zDHHC5 changed in the same manner as palmitoylation of NCX1, suggesting additional regulatory mechanisms may be involved. This study provides novel evidence that palmitoylation of cardiac substrates is altered in the setting of HF, and that expression of zDHHC5 is dysregulated in both hypertrophy and HF.

## Introduction

Heart failure (HF) represents a major economic and global burden affecting over 26 million people worldwide, with ∼3.6 million patients newly diagnosed every year (Ambrosy et al., 2014; Simmonds et al., 2020). All clinical therapies currently aim to reduce myocardial demand by either reducing peripheral resistance and pre-load [i.e., angiotensin-converting enzyme inhibitors (ACEi)] or ventricular after-load and remodelling (i.e., β-blockers; Shah A. et al., 2017). This largely helps to compensate for reduced ejection fraction and systolic dysfunction [as seen in HF with reduced ejection fraction (HFrEF)] and is therefore more effective for management of HFrEF than for HF with preserved ejection fraction (HFpEF). However, even though improved therapeutic recommendations for HFrEF significantly reduce morbidity and mortality rates in clinical trials, prognosis is still poor in this group with a trial of 40,000 hospitalised HF patients demonstrating a 5-year mortality rate of 75%, independent of LVEF (McMurray et al., 2014; Shah K. S. et al., 2017). As such, there remains a pressing and largely unmet clinical need for HF therapies.

Understanding the molecular basis of HF, in both humans and animal models, is essential to developing and directing appropriate therapies. However, this task is complicated by the fact that compensatory cardiac hypertrophy, leading to normal or even enhanced systolic function and left ventricular (LV) enlargement, often precedes decompensated HF and eventual LV dilatation and dysfunction (Kemp and Conte, 2012). The biochemical and molecular changes in both these phases of the disease are distinct, and currently available animal models are often better at modelling hypertrophy than the complex clinical syndrome of HF (Houser et al., 2012). Interestingly, a key component of cardiac dysfunction associated with both cardiac hypertrophy and HF is the dysregulation in expression, modification and function of proteins involved in excitation contraction coupling (ECC). Many of the pathological changes in protein function in ECC during cardiac disease can be attributed to changes in post-translational modification (PTM) regulation, including phosphorylation (Ramila et al., 2015; Sequeira and Maack, 2018) and redox modifications like S-glutathionylation (Pastore and Piemonte, 2013). The majority of proteins involved in ECC have been reported to be regulated by the lipid post-translational modification palmitoylation (see review by Essandoh et al., 2020) with recent proteomic analysis suggesting over 450 cardiac proteins are palmitoylated (Miles et al., 2021). Palmitoylation is a reversible modification involving the attachment of a fatty acid (most commonly palmitoyl derived from palmitoyl-CoA) to the thiol of a cysteine residue. This has a wide range of physiological effects, including regulating the location of the palmitoylated substrate, altering its conformation and consequently activity, or regulating protein-protein interactions. The modification is catalysed by a group of structurally related zDHHC-palmitoyl acyltransferases (zDHHC-PATs) which are integral membrane proteins located throughout the secretory pathway (Main and Fuller, 2021).

Although important in regulating ECC substrate activity, the role of palmitoylation in cardiac disease and HF remains largely unknown. There have been limited studies conducted in zDHHC knock-out animals, but zDHHCs show redundancy and therefore determining individual enzyme characteristics *via* this route is challenging (Main and Fuller, 2021). The most well characterised cardiac zDHHC-PAT is cell surface localised zDHHC5 (see review by Woodley and Collins, 2021). NCX1 and Na^+^/K^+^ ATPase both interact with zDHHC5 after its fourth transmembrane domain, in a site that also interacts with its accessory proteins Golga7 and Golga7b (Ko et al., 2019; Plain et al., 2020; Woodley and Collins, 2021). zDHHC5 directly palmitoylates ECC substrates NCX1 and PLM (Howie et al., 2014; Gök et al., 2020). ZDHHC5 has attracted attention in cardiac physiology as it is a key contributor to massive endocytosis (MEND) of the cellular membrane in anoxia/reperfusion (A/R) injury, with zDHHC5 knock-out hearts showing enhanced functional recovery from A/R injury (Hilgemann et al., 2013; Lin et al., 2013). In the present study, we evaluated changes in zDHHC5 expression in animal models of left ventricular hypertrophy and heart failure, as well as in human ischaemic heart failure samples. We provide the evidence that palmitoylation of cardiac substrates is altered in the setting of HF, and that expression of zDHHC5 is dysregulated in both hypertrophy and HF.

## Materials and methods

### Human organ donor and ischaemic heart failure samples

Samples from organ donors (non-failing) and human heart failure patients were obtained from the Gill Cardiovascular Biorepository at the University of Kentucky, of which patients and families of organ donors provided written consent. All procedures were approved by the local IRB and details of the collection procedures have been published previously (Blair et al., 2016). The study conformed with the principles in the Declaration of Helsinki. Samples in this study were taken from the ventricular endocardium of each heart with details in Supplementary Table S1.

### Mouse model of cardiac hypertrophy induced by pressure overload

Ventricular hypertrophy was induced by pressure overload *via* aortic constriction in 6-week old C57BL/6J mice, as has been previously described (Boguslavskyi et al., 2014).

### Porcine model of heart failure

Pigs were subjected to left anterior descending artery balloon occlusion to induce a myocardial infarction, as has been described previously (Tilemann et al., 2013). Tissue was harvested for molecular analysis after the development of heart failure (3 months post-MI) along with sham controls.

### Ethical statement

Animals were handled in accordance with the UK Animals (Scientific Procedures) Act of 1986. All procedures were approved by the UK Home Office (PP7088535) and Glasgow University Ethics Review Committee. The animal studies were reviewed and approved by the Animal Welfare and Ethical Review Body, University of Glasgow and the Animal Welfare and Ethical Review Body, King’s College London.

### Rabbit model of myocardial infarction and heart failure

Adult male New Zealand White rabbits (12 weeks old, ∼3–4 kg) were given premedication with 0.4 ml/kg intramuscular Hypnorm (fentanyl citrate, 0.315 mg/ml: fluanisone 10 mg/ml, Janssen Pharmaceuticals). Anaesthesia was induced with 0.25–0.5 mg/kg midazolam (Hypnovel, Roche) *via* a cannula in the marginal ear vein. Rabbits were intubated and ventilated using a Harvard small animal ventilator with a 1:1 mixture of nitrous oxide and oxygen containing 1% halothane at a tidal volume of 50 ml and a frequency of 40 min^−1^. Preoperative antibiotic prophylaxis was given with 1 ml Amfipen (ampicillin 100 mg/ml, Mycofarm UK Ltd.) intramuscularly. A left thoracotomy was performed through the fourth intercostal space. Quinidine hydrochloride (10 mg/kg; Sigma Pharmaceuticals), a class IA antiarrhythmic (potassium channel blocker) was administered intravenously prior to coronary artery ligation to reduce the incidence of ventricular fibrillation. The marginal branch of the left circumflex coronary artery, which supplies most of the LV free wall, was ligated to produce an ischaemic area of 30%–40% of the LV (Nisbet et al., 2016). Animals were maintained for 8 weeks during which ischaemic cardiomyopathy developed (confirmed by echocardiography measurement) before cells were isolated for molecular analysis.

### Rabbit cardiomyocyte isolation

Adult rabbit ventricular myocytes (ARVM) were isolated from male New Zealand white rabbits as previously described. Isolation of adult rabbit ventricular cardiomyocytes (ARVM) was completed as described previously (Kettlewell et al., 2013). Briefly, New Zealand White male rabbits (12 weeks old, ∼3–4 kg) were euthanised with a terminal dose of sodium pentobarbital (100 mg/kg) with heparin (500IU), following which the heart was removed and retrogradely perfused on a Langendorff system. Enzymatic digestion of the tissue using a protease and collagenase solution occurred for ∼15 min before heart was removed from the system and cut into sections (left atria, right atria, right ventricle, left ventricle and septal regions) and each was finely dissected in Krafte-Brühe solution. The mixture was then triturated and agitated for 10 min before filtering and the cell suspension was centrifuged manually for a minute before the pellet was re-suspended in fresh KB. For experiments, cells were stepped up to physiological calcium in modified Krebs-Henseleit solution, initially containing 100 µM of CaCl_2_ and left to settle for before the process was repeated using 200 µM, 500 µM, 1 mM, and 1.8 mM concentrations of CaCl_2_. Cells were then snap frozen and kept at -80°C or used for functional experiments.

### Viral infection

Adenoviruses expressing zDHHC5 and zDHHS5 were produced in house using the AdEasy system (Agilent). Rabbit ARVM were infected for 18–24 h by adding virus directly to the culture medium.

### Sucrose gradient fractionation

Rabbit cardiomyocytes were homogenised 18–24 h post-infection in 0.5 M Na_2_CO_3_ (pH11) containing Protease Inhibitor Cocktail Set III (1:1,000; #539134, Calbiochem). Samples were sonicated at 5 µm amplitude 3 times in 20 s on/off bursts. The cell lysates were then mixed with an equal volume of 90% sucrose in MES-buffered saline (MBS, 50 mM MES buffer with 1.5 M NaCl, pH 6.5) to make a 45% sucrose component, and 4 ml of this solution was then added to the bottom of a polypropylene centrifuge tube. To this, a layer of 4 ml 35% sucrose in MBS was added by slowly pipetting down the side of the tube, followed by an additional layer of 4 ml 5% sucrose in MBS in a similar fashion. The samples were then centrifuged in a Beckman Optima XL-80k Ultracentrifuge using a SW40.1Ti swinging bucket rotor at 39000RPM for 18 h at 4°C. Following centrifugation, twelve 1 ml fractions were removed from each centrifuge tube. Fractions 4-5 are reported to contain buoyant caveolae membranes where zDHHC5 and Caveolin-3 predominantly localise (Howie et al., 2014), therefore these were combined and the membranes pelleted at 100,000 g for 60 min, and pellet resuspended in SDS-PAGE loading buffer. The same method was used to combine non-caveolae membrane (fractions 8–12).

### Acyl-resin assisted capture

Acyl-resin assisted capture was used to purify palmitoylated proteins in a sample and adapted from a method published previously (Forrester et al., 2011). Cultured and pelleted cells were lysed in blocking buffer containing 1% methyl methanethiosulfonate (MMTS) to methylate free cysteines. Proteins were then precipitated using acetone and the resulting pellet subsequently washed using 70% acetone to remove excess MMTS. The pellets were then re-suspended in binding buffer before a portion of the sample was taken (unfractionated sample, total protein). To the remaining solution, 250 mM NH_2_OH [hydroxylamine (HA), pH 7.5] was added to hydrolyse thioester bonds, with the same concentration of sodium chloride (NaCl, pH 7.5) added in its place for negative control samples. The proteins with free cysteines were the purified using thiopropyl sepharose beads. Palmitoylation of substrates was determined by relative quantity in HA samples compared to unfractionated.

### Immunoblotting

Standard western blotting was carried out using 6%–20% gradient gels. The primary antibodies used were as follows: zDHHC5 (1:1,000, Sigma, HPA014670), NCX1 (1:1,000, Swant, R3F1), PLM (FXYD1, 1:1,000, Abcam, ab76597), Flotillin-2 (1:1,000, BD Biosciences, 610383), Caveolin-3 (1:4,000, BD Biosciences, 610420), HA-tag (1:5,000, Roche, 11867423001). The secondary antibodies used were as follows: Rabbit anti-mouse HRP (1:2,000, Jackson ImmunoResearch 111-035-144), Goat anti-rabbit HRP (1:2,000, Jackson ImmunoResearch 315-035-003), Goat anti-rat HRP (1:2,000, Jackson ImmunoResearch, 313-035-003), Donkey anti-guinea pig (1:2,000, Jackson ImmunoResearch, 106-035-003).

### Confocal microscopy

Cardiomyocytes on 16 mm glass coverslips were fixed with 4% paraformaldehyde (PFA) for 15 min at room temperature. Cells were permeabilised with 0.1% Triton-X100 in PBS for 10 min at room temperature before blocking with 3% BSA in PBS for at least 1 h. Coverslips were incubated with HA-tag primary antibody (1:200, Roche, 11867423001) in 0.1% BSA in PBS for 1 h before subsequent incubation with anti-rat Alexa Fluor 546 secondary antibody (1:400, Thermofisher, A-11081) for an additional hour. Coverslips were then mounted onto glass slides using Dako Fluorescence Mounting Medium with 1 µl/ml 4′,6-diamidino-2-phenylindole (DAPI). Cells were then visualised using a Zeiss LSM 510 META Confocal Microscope with a ×40 objective.

### Contractility measurements

CellOPTIQ^®^ is an *in vitro* system designed by Clyde Biosciences which allows measurements of contractility, voltage and calcium to be carried out in individual adult cardiomyocytes (Clyde Biosciences Ltd.; Glasgow, UK). Following culture and infection of cardiomyocytes for 18–24 h, cells were transferred to a modified Krebs-Henseleit solution containing 1.8 mM CaCl_2_ and incubated at 37°C using a heated stage. Cells were paced using electrodes with 40 V pulses of 0.2 ms duration at a frequency of 2 Hz. Five second recordings at 100 fps were then taken of individual cells using a ×60 objective. Contractility recordings were analysed using an ImageJ Macro prepared by Dr Francis Burton which analyses the changes in sarcomere length as a measurement of cardiomyocyte contractility by determining the spatial frequency of the intensity profile of sarcomere bands over time. Each recording produced roughly 10 contractile peaks which were averaged to give one trace per cell which was analysed for contractility parameters of amplitude, time to up and down 90% of peak, contractile duration at 50% and 90% of peak and overall time to peak (Rocchetti et al., 2014).

### Statistics

Statistical analysis was completed using GraphPad Prism (Version 7; California, United States) and was performed on groups with three biological replicates or more. For comparisons in data sets with more than two groups, a one-way analysis of variance (ANOVA) with a Sidak’s or Dunnett’s post-hoc test was used, with comparisons detailed in the figure legend. For comparisons of two groups, a paired or unpaired Student’s *t*-test was used. All samples were tested for the presence of significant outliers (Rout’s test). A probability of *p* < 0.05 was considered to be statistically significant.

## Results

### Remodelling of the cellular palmitoylation machinery in heart failure

**FIGURE 1:**
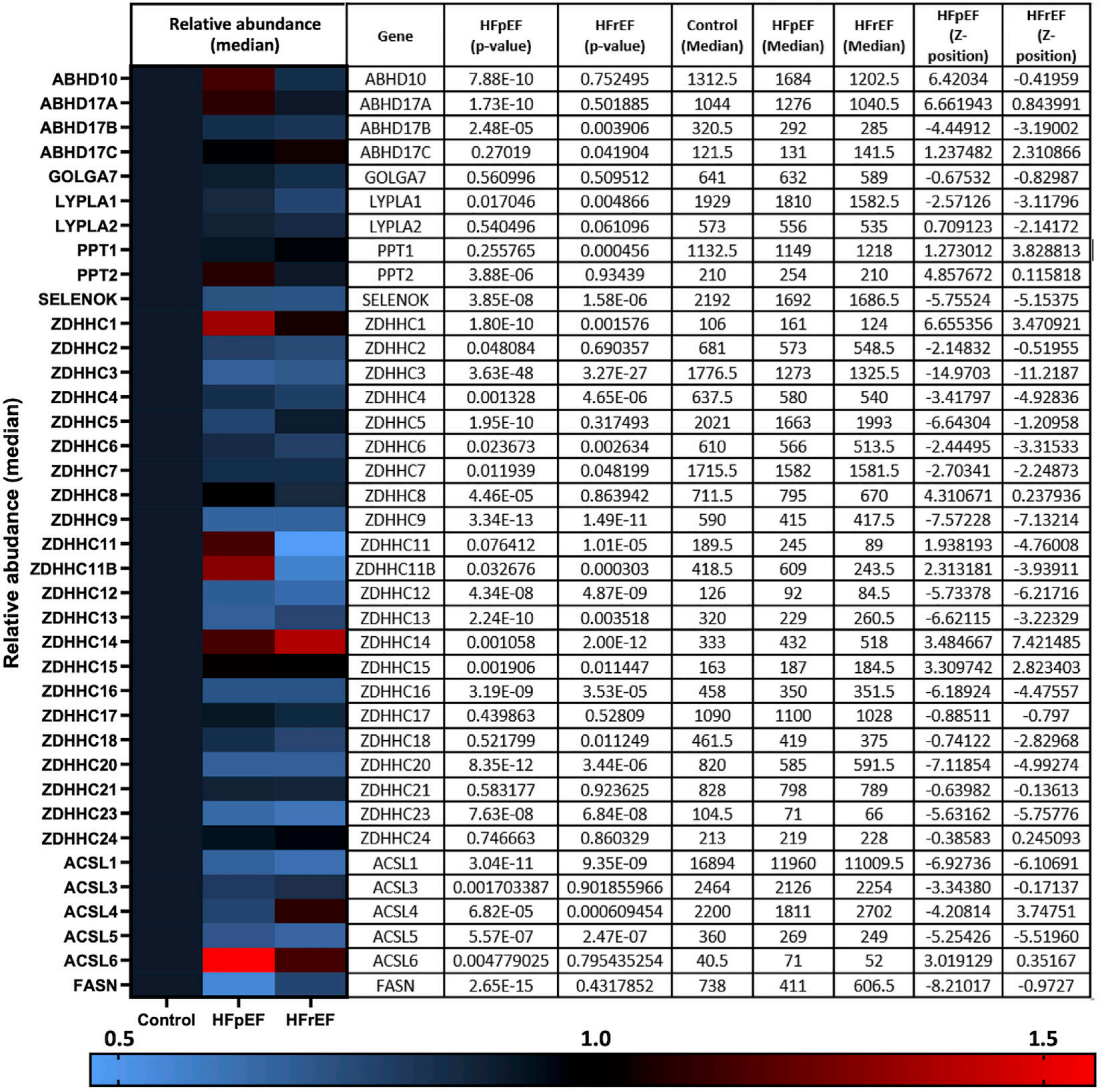
RNA transcript changes of palmitoylating and depalmitoylating enzymes and accessory proteins in heart failure. Hahn and Knutsdottir et al. (2020) performed RNA sequencing on biopsies from heart failure patients with preserved ejection fraction (HFpEF, *n* = 41), reduced ejection fraction (HFrEF, *n* = 30) and donor controls (*n* = 24). Abundance of transcripts of palmitoylating and depalmitoylating enzymes, including 23 zDHHC-palmitoyl acyltransferases, as well as accessory proteins and proteins involved in fatty acid and fatty acyl-CoA synthesis, were investigated in the available data. HFpEF and HFrEF are plotted relative to control (normalised to 1) in the heat map with relative abundance and *p*-values detailed in the accompanying table produced using the data repository from Hahn et al. (2021).

There remains a pressing need to understand the gene and protein expression changes in the failing myocardium in HFpEF and HFrEF in order to tailor existing, and develop new, therapeutic options. Despite the knowledge that zDHHC5 is involved in A/R injury and regulates activity of several important cardiac substrates, the expression or activity of zDHHC5, or any other palmitoylating or depalmitoylating enzymes, has not been investigated in a HF setting. Recently, Hahn and Knotsdottier et al. completed a comprehensive study of RNA transcript changes in HFpEF and HFrEF compared to organ donor controls. To investigate the relevance of palmitoylation as a modification in heart failure, using the available data, we plotted the change in relative abundance in HFpEF and HFrEF compared to organ donor controls of all available palmitoylating enzymes (zDHHC-PATs), depalmitoylating enzymes (LYPLAs, PPTs and ABHDs), accessory proteins [Selenoprotein-K (Fredericks et al., 2017) and Golga7 (Ko et al., 2019)] and proteins involved in fatty acyl CoA production [acyl-CoA synthases (ACSL) and fatty acid synthase (FASN)]. Several of these were found to be both up and down regulated in the failing myocardium, although many to a modest extent, with some changes unique to one phenotype. This included zDHHC5 which was significantly reduced in HFpEF but not HFrEF. Fatty acid and fatty acyl CoA availability have recently emerged as regulators of protein palmitoylation (Main and Fuller, 2021). For example, acyl-CoA synthase (ACSL) isoforms physically associate with zDHHC5, and acyl-CoA synthase activity is required for insulin-induced palmitoylation of NCX1 (Plain et al., 2020; Gök et al., 2022). ACSLs 1 and 4 were significantly downregulated in both HFpEF and HFrEF, with significant changes in all other isoforms and fatty acid synthase in HFpEF only (Figure 1; Hahn et al., 2021).

### zDHHC5 expression and substrate palmitoylation in cardiac hypertrophy

**FIGURE 2:**
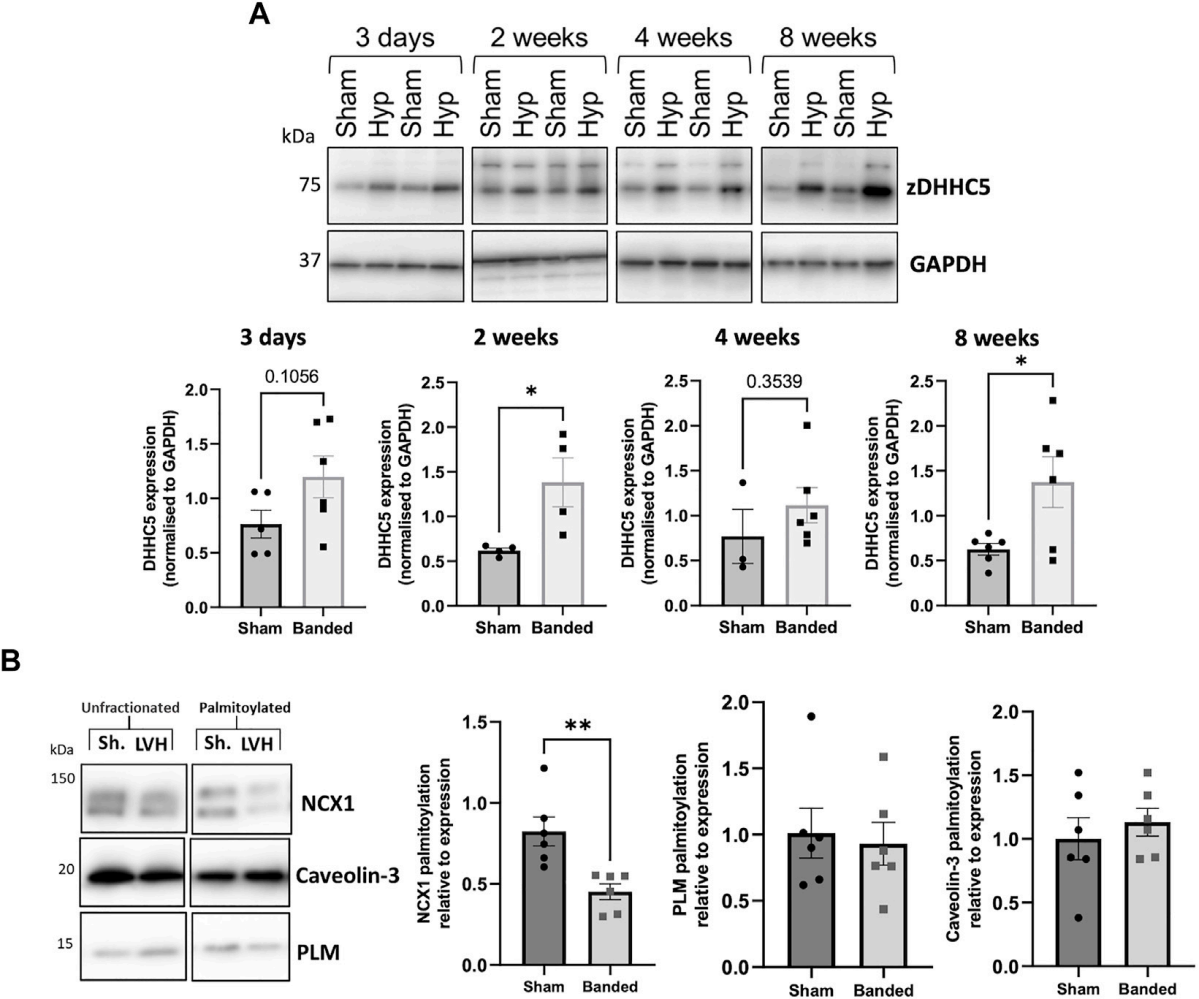
**(A)** Expression of zDHHC5 is increased in mice with left ventricular hypertrophy 2 weeks and 8 weeks post-onset and palmitoylation of NCX1 is reduced at 8-week. Ventricular samples from mice that developed left ventricular hypertrophy (LVH) induced by pressure overload and sham controls were taken at 3 days, 2 weeks, 4 weeks, and 8 weeks post-onset. Expression of zDHHC5 was significantly increased in hypertrophy samples compared to control at 2 weeks and 8 weeks post-onset. **(B)** Acyl-RAC of samples taken at 8-week post injury revealed palmitoylation of zDHHC5 substrate NCX1 was significantly reduced whilst palmitoylation of PLM remained unchanged. zDHHC5 expression is normalised to loading control GAPDH. Statistical comparisons made by unpaired Student s t-test. Data are mean ± S.E.M. *p < 0.05, **p < 0.01.

Cardiac hypertrophy and associated remodelling of the left ventricle precedes many forms of cardiac disease, including heart failure, and is recognised as a crucial step in its pathophysiology (Rame and Dries, 2007). Left ventricular hypertrophy (LVH) induced by pressure overload *via* aortic constriction (“banding”) is commonly used to investigate early molecular changes associated with pathological remodelling. This mouse model (described in Boguslavskyi et al., 2014) displays a 50% increase in LV mass 2 weeks after banding the thoracic aorta and reduced ejection fraction 8 weeks after surgery. We investigated changes in zDHHC5 expression associated with the onset of LVH. Compared to sham operated control mice, zDHHC5 expression was modestly increased from the earliest timepoint investigated (3 days post-banding) and was significantly elevated 2-week and 8-week after surgery. Unexpectedly, whilst zDHHC5 expression was increased 8-week post injury, palmitoylation of zDHHC5 substrate NCX1 was significantly reduced, whilst palmitoylation of its substrate PLM remained unchanged (Figure 2).

**FIGURE 3:**
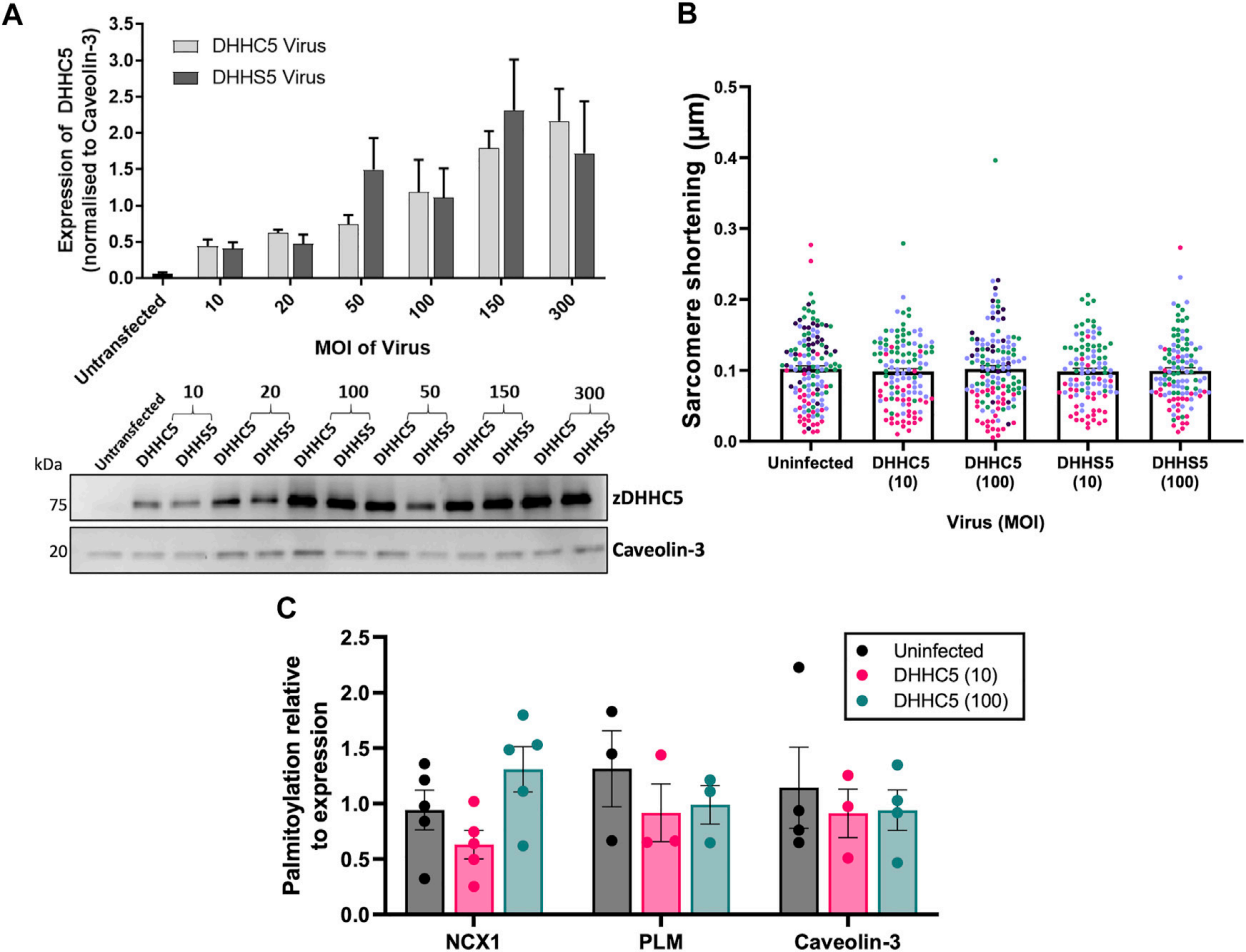
zDHHC5 overexpression does not alter parameters of rabbit ventricular cardiomyocyte contractility or lead to changes in substrate palmitoylation. **(A)** Rabbit ventricular cardiomyocytes were cultured for 18–24 h in the presence of either the HA-DHHC5 virus or a HA-DHHS5 dominant-negative virus, at an increasing range of virus particles per cell or multiplicity of infection (MOI; 10, 20, 50, 150, 300). zDHHC5 protein expression was determined *via* western blot, normalising to housekeeper protein Caveolin-3. HA-DHHC5 and HA-DHHS5 viral infection led to a dose-dependent increase in zDHHC5 expression. Data are expressed as mean ± S.E.M and is a representative image of *n* = 3–4 biological replicates. **(B)** Contractility recordings from cells infected with MOI of 10 and 100 were taken using CellOPTIQ^®^. Viral infection with either zDHHC5 or zDHHS5 at MOI 10 or 100 had no significant effect on altering the parameters of contractility including sarcomere shortening. *N* = 3 biological replicates for S10, C100, S100 and *n* = 4 biological replicates for uninfected and C100, with *n* = 20–51 cells per replicate. Each colour represents cells from one biological replicate. **(C)** Rabbit ventricular cardiomyocytes overexpressing HA-zDHHC5 (MOI 10 and 100) showed no significant change in NCX1 or PLM palmitoylation. Data are mean ± S.E.M analysed *via* an unpaired *t*-test (LVH) and a one-way ANOVA with a Dunnett’s post-hoc test (overexpression) or a Sidak’s post-hoc test (substrate palmitoylation). *p < 0.05.

As elevated zDHHC5 expression but paradoxically reduced levels of NCX1 palmitoylation were associated with the development of LVH, we investigated whether increasing zDHHC5 expression in cardiomyocytes could be directly contributing to the contractile dysfunction observed in this phenotype. We engineered adenoviruses expressing HA-tagged zDHHC5 and catalytically inactive zDHHS5, and infected adult rabbit ventricular cardiomyocytes, achieving dose dependent increases in zDHHC5 expression levels (Figure 3A). Confocal microscopy revealed localisation of HA-tagged zDHHC5 in intercalated discs, cell surface and perinuclear membrane, whilst sucrose gradient fractionation indicated that virally encoded HA-zDHHC5 localised to buoyant membranes prepared using sucrose gradients alongside Caveolin-3, in a similar manner to endogenous zDHHC5 (Howie et al., 2014; Supplementary Figure S1). Changes in contractile function were investigated using the CellOPTIQ^®^ contractility system (Clyde Biosciences Ltd.), and contractility parameters measured by determining the spatial frequency of the intensity profile of sarcomere bands over time (Supplementary Figure S2; Rocchetti et al., 2014). Overexpression of zDHHC5 or zDHHS5 had no effect on contractile force (as determined by sarcomere shortening (Figure 3B) or on any other parameters of contractility (Supplementary Figure S3). Viral overexpression of zDHHC5 did not lead to changes in palmitoylation of its substrates NCX1 or PLM (Figure 3C).

### zDHHC5 expression and substrate palmitoylation in heart failure

**FIGURE 4:**
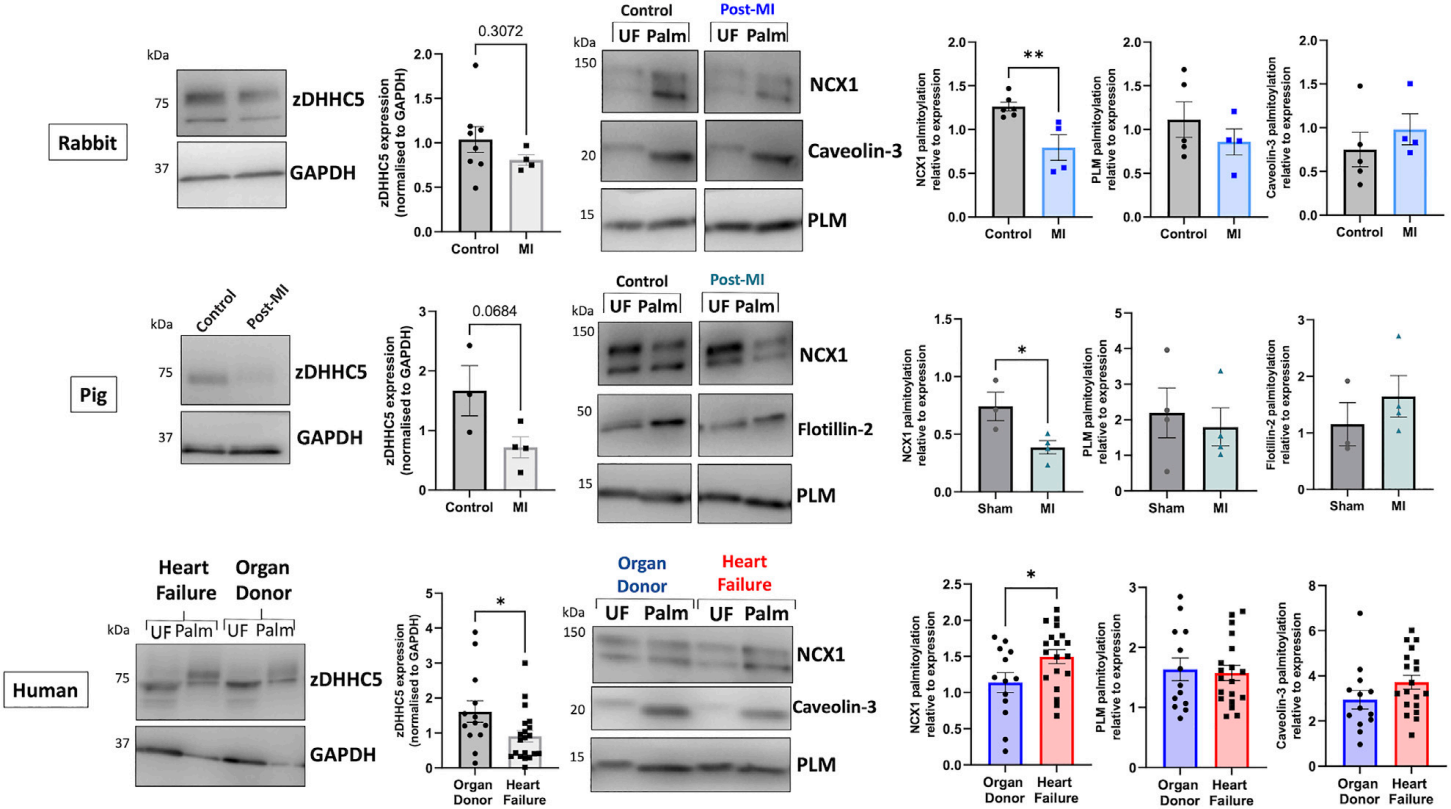
zDHHC5 expression and substrate palmitoylation in heart failure. In the rabbit model of MI-induced HF (8-week post-MI), zDHHC5 expression was unchanged whilst NCX1 palmitoylation was significantly reduced with no change in PLM or Caveolin-3 palmitoylation. In the pig model of MI-induced HF with reperfusion (3-month post-MI) zDHHC5 expression was modestly reduced (*p* = 0.0684) whilst NCX1 palmitoylation was significantly reduced with no change in PLM or Flotillin-2 palmitoylation. In samples from patients with ischaemic heart failure, zDHHC5 expression was significantly reduced compared to organ donor controls whilst NCX1 palmitoylation was significantly increased with no change in PLM or Caveolin-3 palmitoylation. zDHHC5 expression was normalised to loading control GAPDH. Data are palmitoylated fraction normalised to total protein (unfractionated, UF). Statistical comparisons made by unpaired Student’s *t*-test. Data are mean ± S.E.M. *p < 0.05, **p < 0.01.

We investigated whether the differences in zDHHC5 expression and substrate palmitoylation identified early in cardiac hypertrophy persists in HF by analysing two experimental models (rabbit and pig) of myocardial infarction (MI) induced HF, as well as samples from ischaemic human HF patients (classified as reduced ejection fraction, details in Supplementary Table S1). In contrast to LVH, zDHHC5 expression was unchanged or modestly reduced in post-MI samples compared to control. Additionally, despite RNA sequencing data suggesting zDHHC5 expression levels are reduced in HFpEF but unchanged in HFrEF (Figure 1; Hahn et al., 2021) we found that protein expression of zDHHC5 was significantly reduced in ischaemic HF samples compared to organ donors. Interestingly, whilst PLM palmitoylation remained unchanged in all cases, NCX1 palmitoylation was significantly reduced in animal models of HF but increased in human heart failure (Figure 4).

### zDHHC5 palmitoylation in heart failure

**FIGURE 5:**
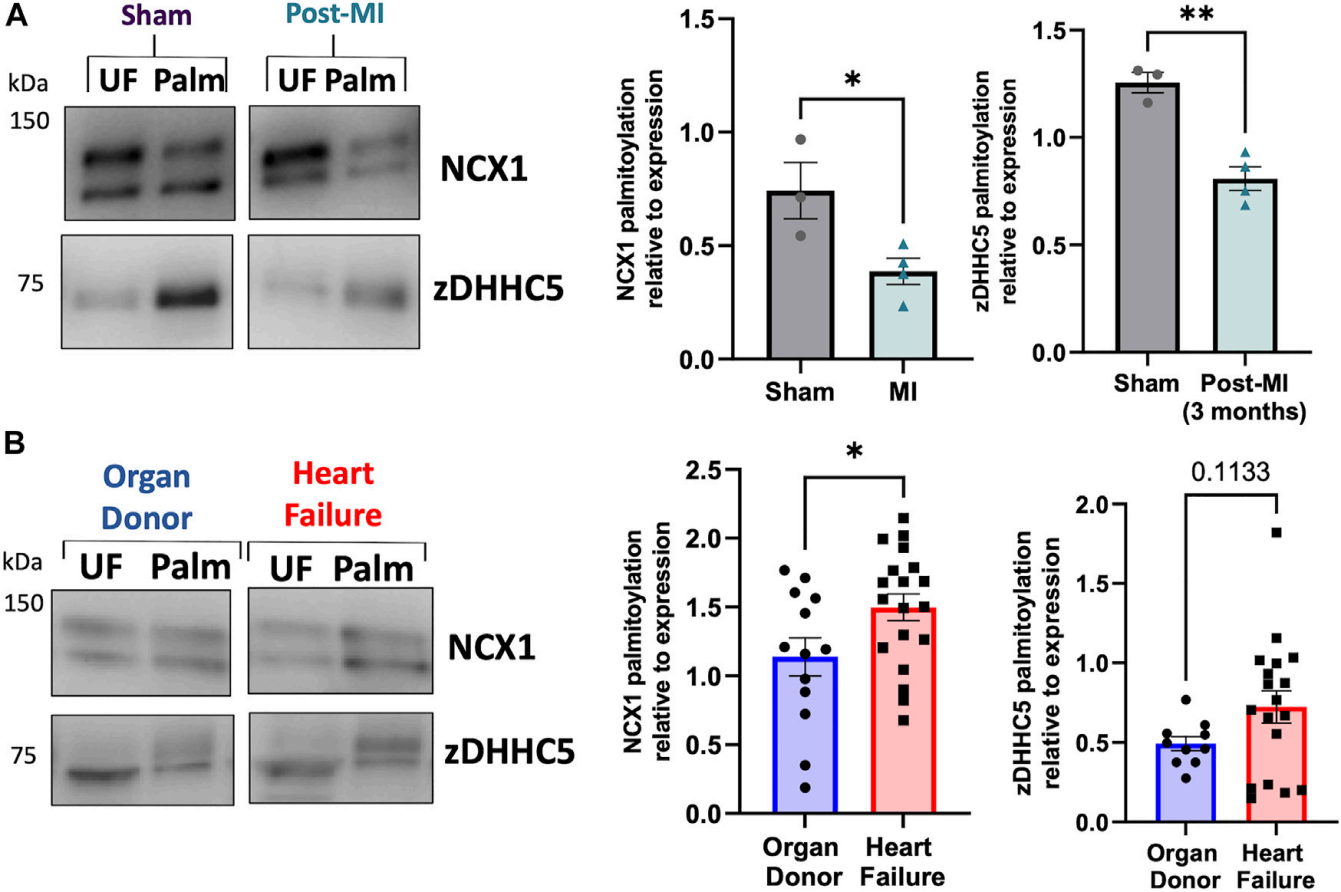
zDHHC5 palmitoylation is altered in heart failure in a similar manner to NCX1 palmitoylation. **(A)** In a pig model of MI-induced heart failure, zDHHC5 palmitoylation is significantly reduced, whilst **(B)** in human heart failure samples, zDHHC5 palmitoylation is modestly increased (*p* = 0.1133). NCX1 data taken from Figure 4 for comparison. Statistical comparisons made by unpaired Student’s *t*-test. Data are mean ± S.E.M. *p < 0.05, **p < 0.01.

The results thus far suggest zDHHC5 expression levels correlate poorly with palmitoylation of its substrates, and that in ventricular muscle changes in zDHHC5 expression alone may not be sufficient to increase changes in palmitoylation of its substrates. ZDHHC5 is the target of PTMs itself, including palmitoylation, which occurs on its active site cysteine during autopalmitoylation before transferring the palmitate to a substrate cysteine (Brigidi et al., 2015). Additionally, zDHHC5 is palmitoylated at additional sites in its C-terminal tail, which is key to mediating its response to β-adrenergic signalling and facilitating its interaction with the Na^+^/K^+^ ATPase, which regulates recruitment and palmitoylation of PLM (Yang et al., 2010; Chen et al., 2020; Plain et al., 2020). As such, we investigated whether palmitoylation of zDHHC5 was changed in HF. Interestingly, zDHHC5 palmitoylation was altered in HF in a similar manner to that of NCX1, whereby zDHHC5 palmitoylation was significantly reduced in the pig model (Figure 5A), but modestly (albeit not significantly) increased in human HF samples (Figure 5B).

## Discussion

**FIGURE 6:**
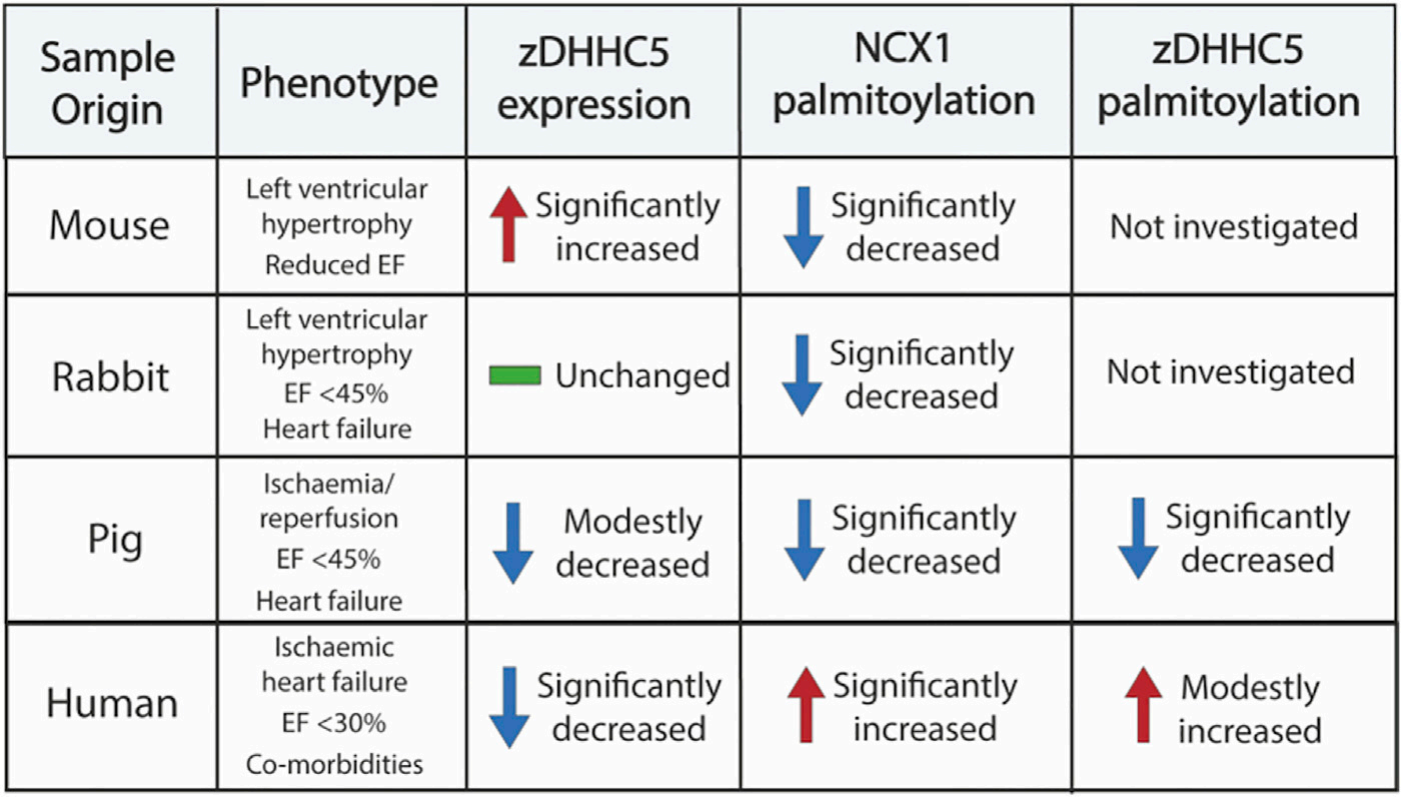
Summary of samples investigated in this study and the changes in zDHHC5 expression and palmitoylation of NCX1 and zDHHC5. Trends or changes are in comparison to relevant controls. EF, ejection fraction.

Palmitoylation has emerged over the last decade a crucial regulatory modification for every class of protein, including several involved in cardiac excitation-contraction coupling (Chien et al., 1996; Tulloch et al., 2011; Howie et al., 2013; Gök et al., 2020; Main et al., 2022). As many of these proteins are dysregulated in diseases such as HF, palmitoylation may represent a novel mechanism to manipulate their function for therapeutic benefit (Main and Fuller, 2021). Despite this, studies investigating changes in palmitoylation in a cardiac disease setting have been limited. As such, we focussed our investigation on the most well classified cardiac zDHHC-PAT, zDHHC5, which has been implicated in A/R injury and regulates the palmitoylation of important ion transporters and accessory proteins in the heart (Tulloch et al., 2011; Hilgemann et al., 2013; Lin et al., 2013; Howie et al., 2014; Chen et al., 2020; Gök et al., 2020; Plain et al., 2020). In the present study, we provide novel evidence that zDHHC5 expression and palmitoylation are altered in cardiac disease, although this does not directly correlate with a change in the palmitoylation of its substrates NCX1 and PLM (summarised in Figure 6).

Firstly, we observed that in a LVH model of cardiac hypertrophy and remodelling, zDHHC5 expression was upregulated as early as 3 days post-onset, and this was maintained until 8 weeks post-injury (Figure 2). Because there was such an acute upregulation of zDHHC5, we investigated the impact of overexpressing zDHHC5 in cardiomyocytes to determine if there was a functional consequence for ventricular contractility, but this had no effect on any contractile parameters investigated (Figure 3; Supplementary Figure S3). In both LVH and virally infected cardiomyocytes, despite increased zDHHC5 expression, there was no correlated increase in substrate palmitoylation, as palmitoylation of PLM remained unchanged, whilst NCX1 palmitoylation was significantly reduced in LVH samples (Figure 3). This suggests that zDHHC5 expression levels are not rate limiting for substrate palmitoylation in ventricular muscle, and implies as-yet unidentified mechanisms control zDHHC5 activity and substrate palmitoylation in the heart. In other settings, availability of palmitoyl-CoA (Lin et al., 2013) and activity of acyl-CoA synthase enzymes (Gök et al., 2022), which may associate with zDHHC-PATs (Plain et al., 2020), have been established to be important determinants of substrate palmitoylation.

We investigated whether the changes in zDHHC5 expression were observed later in disease pathogenesis using models of ischaemic HF (rabbit and pig), as well as samples from ischaemic HF patients. In contrast to cardiac hypertrophy, zDHHC5 expression was either unchanged (rabbit), modestly reduced (pig) or significantly reduced (human) in a HF setting. However, similarly to the LVH model, this was poorly correlated with changes in substrate palmitoylation, such that palmitoylation of NCX1 was significantly reduced in the animal models of HF, but was significantly increased in human HF samples (Figure 4). Throughout this investigation we observed changes in zDHHC5 expression that did not consistently match changes in palmitoylation its substrates NCX1 and PLM. Indeed in the largest cohort examined (human HF and organ donors), no relationship between zDHHC5 expression levels and substrate palmitoylation levels can be detected (Supplementary Figure S4).

ZDHHC5 itself is under the control of several regulatory pathways, including palmitoylation of cysteines in its C-terminal tail which has important functional consequences for substrate recruitment and palmitoylation (Chen et al., 2020; Plain et al., 2020). In addition, its activity may be controlled by the availability of its substrate acyl-CoA, synthesised by ACSL isoforms. Analysis of zDHHC5 palmitoylation revealed that, similar to NCX1, palmitoylation was significantly reduced in the pig model and modestly increased (although not significantly) in the human heart samples (Figure 5). This may suggest there are upstream regulatory pathways driving changes in substrate palmitoylation, including that of zDHHC5. ZDHHC-PATs have been frequently reported to palmitoylate each other, and a proximity biotinylation screen identified DHHC20 as an interactor and palmitoylating enzyme of zDHHC5. Palmitoylation of the zDHHC5 C-terminal tail in response to adrenergic stimulation is required for its own stabilisation at the plasma membrane (Chen et al., 2020; Woodley and Collins, 2021). The zDHHC5 palmitoylation sites lie in an amphipathic helix containing a binding site for the Na^+^/K^+^ ATPase and zDHHC5 accessory protein GOLGA7, which controls its membrane localisation (Woodley and Collins, 2019; Plain et al., 2020). Increased zDHHC5 palmitoylation as observed in human HF, or reduced palmitoylation as observed in the pig model, may suggest increased or decreased activity of zDHHC5 either through a change in palmitate loading into the active site or a change in zDHHC20-mediated palmitoylation. We also do not rule out the possibility that the mismatch between zDHHC5 expression and palmitoylation of its substrates is caused changes in zDHHC5 subcellular localisation. Localisation-dependent palmitoylation of zDHHC5 substrates has previously been demonstrated following increased neuronal activity (Brigidi et al., 2015). Although we observe the virally-expressed zDHHC5 to be correctly localised to caveolar membranes in the cells surface (Supplementary Figure S1), it cannot be ruled out that altered zDHHC5 subcellular localisation in disease may change its ability to locate and palmitoylate its substrates. It should also be noted that observation of zDHHC5 expression and substrate palmitoylation in tissue samples (mouse, pig and human) may include a contribution of non-myocyte cells [although cardiomyocytes represent ∼75% of myocardial tissue volume (Vliegen et al., 1991)].

The impact of other zDHHC5 PTMs on its activity will be important to consider in future investigations, as phosphorylation has been observed to inactivate zDHHC5 (Hao et al., 2020; Schianchi et al., 2020), while O-GlycNAcylation of zDHHC5 enhanced PLM association and palmitoylation (Plain et al., 2020; Schianchi et al., 2020). In line with our observations from hypertrophic tissue, but in contrast to the ischaemic human HF results, Miles et al. (2021) report significantly increased levels of zDHHC5 in human HF. However, the samples investigated represent a mixture of ischaemic and non-ischaemic origin (Miles et al., 2021), limiting the value of a comparison to the ischaemic samples used in our investigation. Nevertheless, given RNA-sequencing suggests levels are significantly reduced in HFpEF but not HFrEF (Hahn et al., 2021), further characterisation of zDHHC5 expression should be carried out in additional cohorts of patients.

Steady state protein palmitoylation is controlled by the balanced activities of palmitoylating and depalmitoylating enzymes. Several depalmitoylating enzymes show altered expression in both HFrEF and HFpEF, although not to the same extent as the dysregulated of zDHHC-PAT expression (Figure 1). In comparison to zDHHC-PATs, relatively little is known about the regulation of these enzymes, although APT1 was recently demonstrated to depalmitoylate NCX1 (Gök et al., 2021). However, we did not find a significant change in APT1 protein abundance in the setting of HF, so this likely does not explain the changes in substrate palmitoylation observed (Supplementary Figure S5).

Aside from information of zDHHC5 expression and palmitoylation, this study provides novel evidence that NCX1 palmitoylation is changed in cardiac disease in animal models and humans. Although NCX1 expression is often increased in the setting of LV dysfunction in HF, this does not necessarily lead to increased NCX1 activity (Hobai and O’Rourke, 2000; Quinn et al., 2003), suggesting that altered post-translational regulation of NCX1 contributes to the remodelling of its activity in HF. Increased NCX1 palmitoylation, as observed in human HF, would enhance its inactivation and reduce Ca^2+^ efflux. Whilst improving systolic function, this would contribute to diastolic impairment, of which NCX1 is a key mediator (Kass et al., 2004). Interestingly, the pattern of NCX1 palmitoylation associated with HF that we report here was recapitulated for cardiac myosin binding protein-C, again suggesting that upstream factors such as a fatty acid and fatty acyl-CoA availability may be responsible for the changes of substrate palmitoylation (Main et al., 2022).

A significant finding, but also a limitation of this study, is the lack of consistency in the changes in zDHHC5 expression and substrate palmitoylation between different animal models and human HF. This makes it challenging to draw definitive conclusion regarding the importance of palmitoylation in cardiac disease. The increased palmitoylation in the human setting may be a result of more developed decompensation compared to the animal models, or as a result of pharmacological intervention. It may also reflect a failure of the animal model to accurately reflect human pathology. Indeed, this is a major limitation of current animal models for HF research, because they rarely include study of novel therapeutic interventions in combination with current optimal clinical care, making translational potential challenging. Interestingly, whilst NCX1 palmitoylation was frequently altered, PLM palmitoylation did not change in any setting (Plain et al., 2020). However, this observation may be as a result of solely characterising palmitoylation using Acyl-RAC. Whilst this method provides a robust mechanism to detect protein palmitoylation, changes in substrate palmitoylation in singly palmitoylated proteins are most likely to be observed using this capture method, as opposed to substrates with multiple palmitoylation sites, where the substrate will still be captured even if only one site remains palmitoylated. Indeed, this may be why NCX1, with one palmitoylation site, is frequently observed to be changed whilst PLM containing two palmitoylation sites did not vary in any disease state (Tulloch et al., 2011; Reilly et al., 2015; Howie et al., 2018). Experimental approaches that measure palmitoylation site occupancy of multiply palmitoylated proteins may provide further insight into the contribution of aberrant protein palmitoylation to the pathogenesis of heart failure.

## Data Availability

The raw data supporting the conclusion of this article will be made available by the authors, without undue reservation.
